# Drug-Coated Balloons versus Everolimus-Eluting Stents in Patients with In-Stent Restenosis: A Pair-Wise Meta-Analysis of Randomized Trials

**DOI:** 10.1155/2020/1042329

**Published:** 2020-01-21

**Authors:** Nina Peng, Wei Liu, Zongzhuang Li, Jun Wei, Xuejun Chen, Wei Wang, Hao Lin

**Affiliations:** ^1^Department of Internal Medicine, Guizhou Orthopedics Hospital, Guiyang, China; ^2^Department of Cardiology, Guizhou Provincial People's Hospital, Guiyang, China

## Abstract

**Objective:**

This study aimed to compare the effectiveness of drug-coated balloons (DCB) with everolimus-eluting stents (EES) in the treatment of in-stent restenosis (ISR) and the differential relative effect of DCB in patients with drug-eluting stents (DES)-ISR and bare metal stents (BMS)-ISR.

**Background:**

The efficiency and safety of DCB and EES need to be assessed for the treatment of ISR.

**Methods:**

A systematic literature search was conducted using PubMed and EMBASE to identify all relevant studies. Angiographic results and clinical events were separately assessed. Subgroup meta-analyses were performed according to the type of restenosed stent.

**Results:**

Six randomized trials with 1134 patients were included. The overall pooled outcomes indicated that DCB was associated with lower minimum lumen diameter (mean difference (*MD*) = −0.17, 95% *CI* = −0.29 to −0.05, *P* = 0.006) and higher target lesion revascularization (risk ratio (*RR*) = 2.38, 95% *CI* = 1.36 to 4.18, *P* = 0.002) than EES. However, the subgroup meta-analyses showed that DCB was inferior to EES only in DES-ISR patients, with lower minimum lumen diameter (*MD* = −0.25, 95% *CI* = −0.37 to −0.14, *P* < 0.001), higher percent diameter stenosis (*MD* = 5.37, 95% *CI* = 1.33 to 9.42, *P* = 0.009), more binary restenosis (*RR* = 2.07, 95% *CI* = 1.20 to 3.58, *P* = 0.009), and higher incidence of target vessel revascularization (*RR* = 2.07, 95% *CI* = 1.22 to 3.50, *P* = 0.007) and target lesion revascularization (*RR* = 2.43, 95% *CI* = 1.28 to 4.22, *P* = 0.002). No differences in angiographic results and clinical events were found between DCB and EES in BMS-ISR patients.

**Conclusions:**

DCB was inferior to EES in DES-ISR and comparable in BMS-ISR in terms of angiographic results and clinical events.

## 1. Introduction

In-stent restenosis (ISR) is one of the main stumbling blocks for stent implantation in patients with coronary artery disease [1]. Although the medicines and stents had advanced prominently, ISR was still remarkable. Repeat revascularization for ISR accounts for 5–10% of patients undergoing percutaneous coronary intervention (PCI) with drug-eluting stents (DES) and for 20–30% after PCI with bare-metal stents (BMS) [1]. Many repeat revascularization strategies were performed in ISR patients, such as balloon angioplasty, BMS implantation, vascular brachytherapy, rotablation, DES implantation, or drug-coated balloon (DCB) angioplasty [2]. Among these strategies, DES implantation and DCB angioplasty are superior to other strategies [2]. The latest myocardial European revascularization guideline recommended DES implantation and DCB angioplasty for the treatment of ISR both of BMS or DES (class I, level A) [2].

Many trials had compared the efficacy and safety of DES and DCB for the treatment of ISR, but the results varied. A network meta-analysis suggests that PCI with everolimus-eluting stents (EES) and DCB angioplasty should be considered for the treatment of any type of coronary ISR [3]. In this network meta-analysis, EES was the most effective strategy for the treatment of ISR, with the lowest risks of restenosis and repeat revascularization compared with other treatments; DCB ranked second in terms of angiographic and clinical effectiveness [3]. However, this network meta-analysis only included two head-to-head comparative trials, RIBS IV and RIBS V, to synthesize the direct result of EES versus DCB. After this network meta-analysis, several trials compared head-to-head EES with DCB for the treatment of ISR with debated results [4–6]. The comparison of EES and DCB for the treatment of ISR remained controversial. Besides, previous studies demonstrated that DCB angioplasty was more effective in BMS-ISR than in DES-ISR. However, the differential relative efficacy between DCB and EES in patients with BMS-ISR and DES-ISR was still unknown.

Therefore, through a pair-wise meta-analysis of all relevant randomized evidence, this study aimed to directly compare DCB with EES for the treatment of ISR. Subgroup meta-analyses were performed to evaluate the differential relative effect of DCB in patients with DES-ISR and BMS-ISR.

## 2. Materials and Methods

The present systematic review and meta-analysis was performed in compliance with the recommendations of the PRISMA statement (Preferred Reporting Items for Systematic Reviews and Meta-Analyses) [7, 8].

### 2.1. Search Strategy

We searched PubMed and EMBASE (up to June 12, 2019) to identify all publications that compared DCB with DES for ISR therapy. The following terms were used by combining with proper logical connectors: “drug-coated balloon,” “drug-eluting balloon,” “drug-eluting stents,” “everolimus-eluting stents,” “randomized,” “randomized,” “randomly,” “in-stent restenosis,” and “coronary restenosis.” Moreover, a manual search was performed by scanning the references of the identified articles to find potentially missing studies by the electronic searches.

### 2.2. Study Selection and Data Collection

The inclusion criteria of the present systematic review and meta-analysis were as follows: (1) a randomized controlled trial (RCT) was mandated, (2) patients were diagnosed with ISR, both for coronary arteries previously treated with BMS and DES, and (3) studies that compared DCB with EES.

The selection of study was strictly in compliance with the inclusion criteria. Two authors (*NP* and *WL*) independently assessed all potentially relevant studies. The selection process was carried out by crude screening to exclude irrelevant studies at the level of the title and abstract, and the remaining articles studies were double-checked by full text to achieve a final decision. A consensus was reached on all eligible studies between the two screening authors. Any discrepancies were resolved by discussion.

Two authors (*ZL* and *JW*) independently extracted all relevant information from eligible studies. A prespecified table that contained the relevant items was used to help with data collection.

### 2.3. Endpoints

In the present systematic review and meta-analysis, the different effects of DCB and EES for ISR therapy in BMS-ISR or DES-ISR patients were assessed. The angiographic results contained in-segment late lumen loss (LLL), in-segment minimal lumen diameter (MLD), percent diameter stenosis, and binary restenosis at follow-up angiography. The clinical events were target vessel revascularization (TVR), target lesion revascularization (TLR), myocardial infarction, and death. If two follow-up durations were reported, we chose the longer period.

### 2.4. Evaluation of Study Quality and Publication Bias

The Collaboration's “Risk of Bias” tool was also used to assess the risk of bias in the included studies.

### 2.5. Data Synthesis and Statistical Analysis

We conducted the present meta-analyses on angiographic results and clinical events of DCB and EES separately. The *I*^2^ statistic was used to test statistical heterogeneity, with values of >50% representing important heterogeneity, then a random-effects model was used to pool the effect sizes; While *I*^2^ ≤ 50% indicated insignificant heterogeneity, and a fixed-effects model was used to pool the effect sizes. Risk ratio (RR) was calculated as the effect size for endpoints with categorical data, and the mean difference (MD) with the 95% confidence interval (CI) was the effect size for endpoints with continuous data. Subgroup meta-analyses were performed according to the type of restenosed stent (BMS-ISR and DES-ISR). We performed sensitive analyses using leaving-one-out approach. Trial sequential meta-analysis (TSA) was performed to assess the false positive errors (or type I errors) and false negative errors (or type II errors). Continuous data are presented as mean ± standard deviation. Categorical data are presented as values and percentages.

All meta-analyses were pooled based on the *Cochrane Handbook for Systematic Reviews of Interventions Version 5.1.0*. Meta*-*analyses were conducted using the Review Manager software (version 5.3), and TSA were conducted using the TSA software (version 0.9.5.10 Beta).

## 3. Results

**Figure 1:**
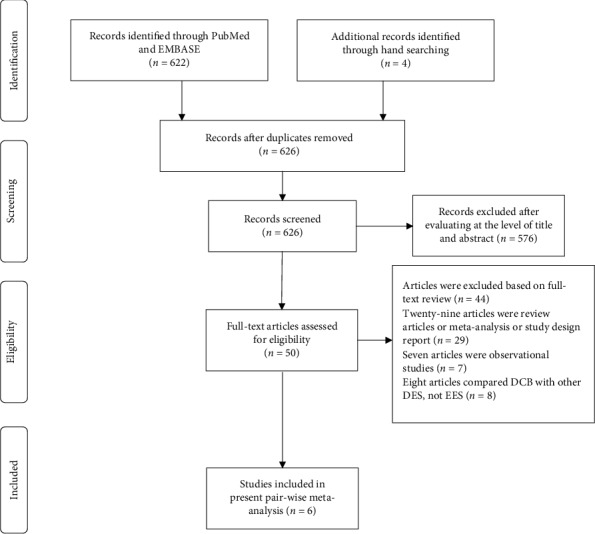
Flow chart of study selection.

A total of 626 potential literature citations matched the systematic search strategy.  Figure 1 presents in detail the study search and selection process. After strict selection, six trials with 1134 patients were included in the present pair-wise meta-analysis [4–6, 9–11]. The TIS and SEDUCE trials were from a single center [6, 10], and the other four trials were performed at multiple centers. The follow-up durations of angiography ranged from 6 to 12 months. All the included trials reported 1-year clinical events, while RIBS IV, RIBS V, and TIS trials further reported 3-year clinical events [6, 9, 11–14]. We used the 3-year follow-up clinical events to synthesize the final forest plots for the three trials [12–14].

The definition of restenosis was >50% diameter stenosis on visual assessment in-stent and/or <5 mm of the stent in all included trials, except for the SEDUCE trial, in which restenosis was defined as >70–<100% for target lesion stenosis measured by quantitative coronary angiography. In the included six trials, the same DCB (paclitaxel-eluting balloon) was used. The clinical and lesions characteristics of included trials are summarized in Tables 1 and 2.

### 3.1. Quality and Risk of Bias of the Included Studies

**Figure 2:**
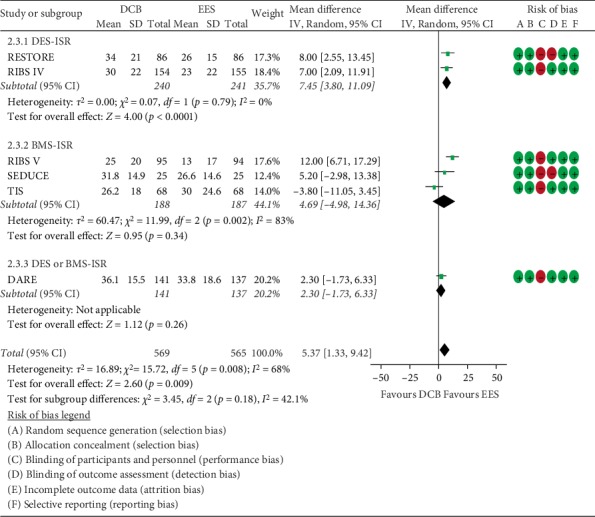
Mean difference in the percent diameter stenosis between the DCB and EES groups, and the risk of bias among included studies. Subgroup analysis was performed based on the type of restenosed stent. BMS, bare-metal stents; DES, drug-eluting stents; DCB, drug-coated balloons; EES, everolimus-eluting stents.

The summary assessment of risk of bias is shown in Figure 2. The quality was “high” because most information was obtained from included RCT studies with low risk of bias.

### 3.2. Angiographic Results at Follow-Up

#### 3.2.1. MLD

All the six trials reported about the MLD. There were 569 patients in the DCB group and 565 patients in the EES group. The overall meta-analysis revealed that EES was superior to DCB in terms of MLD with MD of −0.17 mm with important heterogeneity (*MD* = −0.17, 95% *CI* = −0.29 to − 0.05, *P* = 0.006, *I*^2^ = 64%, Table 3). In the subgroup meta-analysis for DES-ISR, compared to EES, DCB was associated with smaller MLD (*MD* = −0.25, 95% *CI* = −0.37 to − 0.14, *P* < 0.001, Table 3). However, for BMS-ISR, MLD did not show significant difference between DCB and EES (*MD* = −0.15, 95% *CI* = −0.39 to 0.09, *P* = 0.22, Table 3). The results remained stable using the leave-one-out approach after omitting any single trial from the analysis. The TSA showed before reaching the expected sample size, the result that EES was superior to DCB in terms of MLD was inconclusive (Data not shown).

#### 3.2.2. LLL

LLL was evaluated in all six trials. The pooled result showed a similar LLL between the DCB group and EES group for ISR therapy, and significant heterogeneity was identified, i.e., between-trial heterogeneity, with *I*^2^ = 87% (*MD* = −0.06, 95% *CI* = −0.23 to 0.10, *P* = 0.46, *I*^2^ = 87%, Table 3). For subgroup meta-analysis, DCB and EES had similar LLL for both DES-ISR and BMS-ISR (*MD* = 0.04, 95% *CI* = −0.12 to 0.20, *P* = 0.61 for DES-ISR; and *MD* = −0.06, 95% *CI* = −0.35 to 0.24, *P* = 0.71 for BMS-ISR, Table 3). Sensitive analysis by leave-one-out approach showed the overall result remained stable of our study. The TSA indicated more studies were needed to verify the result (Data not shown).

#### 3.2.3. Percent Diameter Stenosis

**Figure 3:**
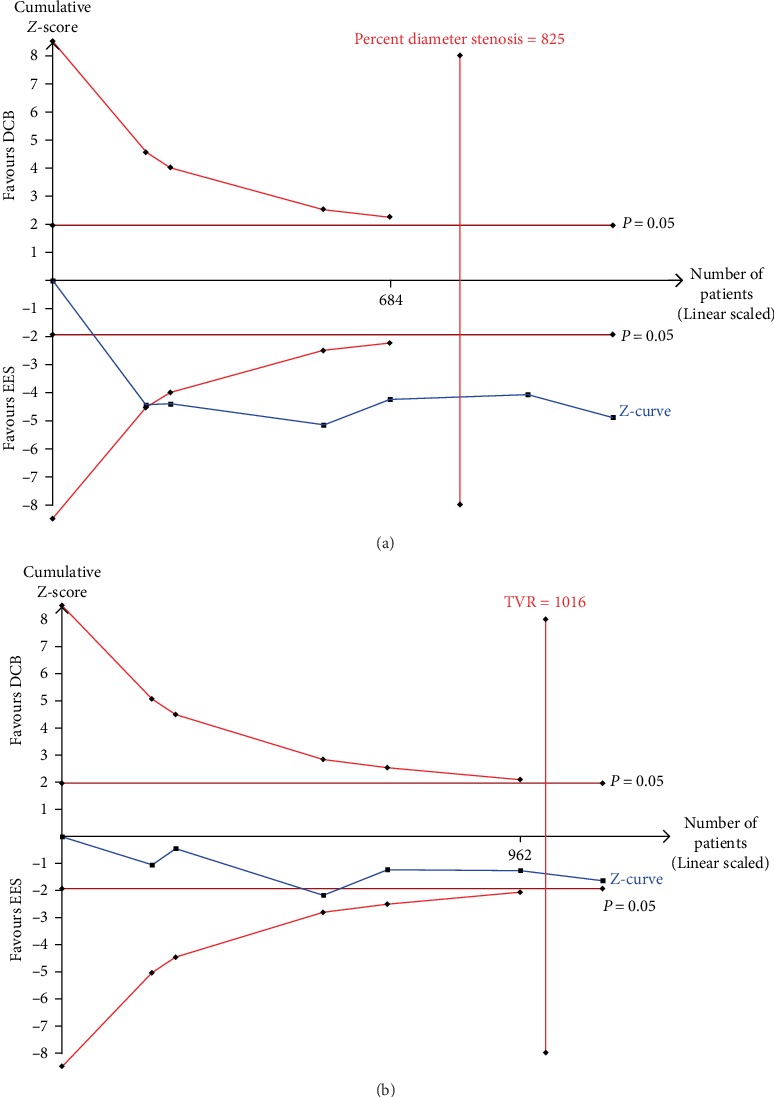
The trial sequential meta-analysis (TSA) of DCB versus EES for the treatment of in-stent restenosis on percent diameter stenosis (a) and target vessel revascularization (b). DCB, drug-coated balloons; EES, everolimus-eluting stents.

All six trials compared the percent diameter stenosis between patients treated with DCB and those treated with EES (569 versus 565 patients, respectively). From the synthetic result, the DCB was associated with higher percent diameter stenosis with a level of MD 5.37 (*MD* = 5.37, 95% *CI* = 1.33 to 9.42, *P* = 0.009, *I*^2^ = 68%, Figure 2). There were significant heterogeneities among trials with *I*^2^ = 68%. For the subgroup meta-analysis, compared with EES, DCB was associated with higher percent diameter stenosis only for DES-ISR (*MD* = 7.45, 95% *CI* = 3.80 to 11.09, *P* < 0.001, Figure 2), but not for BMS-ISR (*MD* = 4.69, 95% *CI* = −4.98 to 14.36, *P* = 0.34, Figure 2). Exclusion of any single trial from the analysis (leave-one-out meta-analysis) did not substantively alter the overall result of our analysis. The TSA indicated the meta-analysis became conclusive according to the O'Brien-Fleming boundaries after the cumulative significance testing (Figure 3(a)).

#### 3.2.4. Binary Restenosis

**Figure 4:**
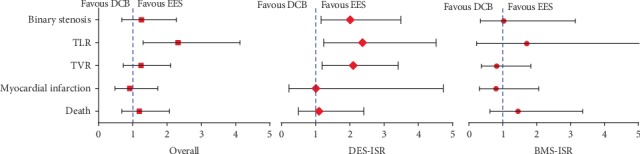
Summary of main event comparisons between DCB and EES across subgroups. BMS, bare-metal stents; DES, drug-eluting stents; DCB, drug-coated balloons; EES, everolimus-eluting stents.

All six trials reported binary restenosis between DCB and EES groups. A total of 70 binary restenosis were identified in the DCB group with 569 patients, while 59 binary restenosis were identified in the EES groups with 565 patients. The pooled result showed that binary restenosis did not differ between the DCB and EES groups (pooled *RR* = 1.25, 95% *CI* = 0.68 to 2.27, *P* = 0.47, *I*^2^ = 58%, Table 3 and Figure 4). The subgroup analyses revealed that the incidence of binary restenosis was 2.01-fold higher in the DCB group for DES-ISR patients (pooled *RR* = 2.07, 95% *CI* = 1.20 to 3.58, *P* = 0.009, Table 3 and Figure 4). While for BMS-ISR patients, the incidence of binary restenosis was similar between the groups (pooled *RR* = 1.03, 95% *CI* = 0.34 to 3.14, *P* = 0.96, Table 3 and Figure 4). The pooled results were robust to the deletion of individual studies, either among the whole group or within each subgroup. The meta-analysis becomes conclusive according to the TSA.

### 3.3. Clinical Events

**Figure 5:**
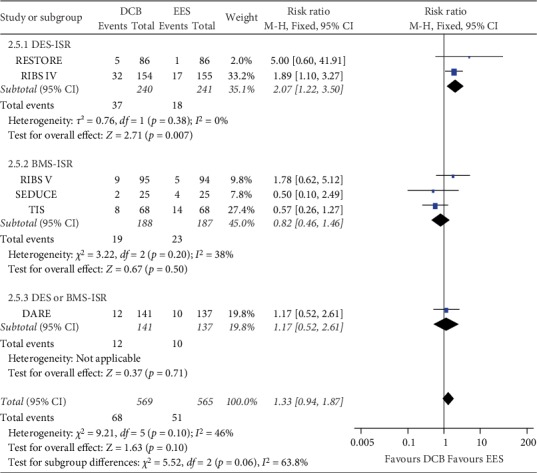
Comparison of the risk of TVR between DCB and EES. Subgroup analysis was performed based on the type of restenosed stent. BMS, bare-metal stents; DES, drug-eluting stents; DCB, drug-coated balloons; EES, everolimus-eluting stents; TVR, target vessel revascularization.

With respect to clinical events, three trials reported 1-year clinical endpoints and another three trials reported 3-year clinical outcomes. All six included studies reported TVR in detail. The overall meta-analysis showed that the incidence of TVR did not differ between the groups (Fixed-effect model *RR* = 1.33, 95% *CI* = 0.94 to 1.87, *P* = 0.44, *I*^2^ = 46%, Figure 5). However, for DES-ISR, there was a 2.07-fold higher TVR in the DCB group than in the EES group (*RR* = 2.07, 95% *CI* = 1.22 to 3.50, *P* = 0.007, Figure 5). For BMS-ISR, the incidence of TVR was comparable between the DCB and EES groups (*RR* = 0.82, 95% *CI* = 0.46 to 1.46, *P* = 0.50, Figure 5). However, the overall result became significant on deletion of the TIS trial (*RR* = 1.61, 95% *CI* = 1.10 to 2.38, *P* = 0.02). The TSA indicated the result is conclusive based on the O'Brien-Fleming boundaries (Figure 3(b)).

Only four trials were eligible for the pooled analysis of TLR. Of 360 patients, 38 (10.6%) patients in the DCB group experienced TLR, while 16 (4.4%) of 360 patients in the EES group received TLR. Pooled analysis using a fixed-effects model showed that DCB had significantly higher TLR than EES (*RR* = 2.38, 95% *CI* = 1.36 to 4.18, *P* = 0.002, *I*^2^ = 0%, Table 3 and Figure 4). Subgroup analyses revealed that DCB increased TLR only in patients with DES-ISR (*RR* = 2.43, 95% *CI* = 1.28 to 4.22, *P* = 0.002, Table 3 and Figure 4) and not in patients with BMS-ISR (*RR* = 2.23, 95% *CI* = 0.70 to 7.10, *P* = 0.17, Table 3 and Figure 4). Sensitivity analysis were not conducted since there are only 4 trials.

No statistically significant differences in myocardial infarction and death in both patients with DES-ISR and BMS-ISR (Table 3 and Figure 4), but more studies were needed to verify the results according to TSA. The direction of the results remained unchanged when removing any single trial.

## 4. Discussion

The present pair-wise meta-analysis included six RCTs with 1134 patients and compared DCB to EES for ISR treatment. The pooled results showed that DCB had differential relative efficacy between DES-ISR and BMS-ISR compared with EES. For DES-ISR, DCB was inferior to EES both on angiographic results and clinical events. In detail, DCB had lower MLD, higher percent diameter stenosis, more binary restenosis, and higher incidence of TVR and TLR than EES in DES-ISR patients. However, in BMS-ISR patients, the efficacy of EES and DCB in terms of angiographic results and clinical events were comparable.

DCB was first introduced in 2006 for the clinical treatment of ISR as it does not require implanting additional metal layers for drug release [15]. Thereafter, many randomized trials attempted to evaluate the efficacy of DCB for the treatment of ISR. Nowadays, robust evidences showed that DCB was superior to uncoated balloon angioplasty for the treatment of DES-ISR and BMS-ISR [16]. With increasing evidences, DCB is an established treatment option of DES-ISR and BMS-ISR with a Class I, Level of Evidence: a recommendation in the European guidelines, the same as DES [2]. However, the efficacy between DCB and DES for the treatment of ISR is still unknown.

Randomized trials have demonstrated that DCB is associated with comparable angiographic results and clinical events with first-generation DES [17, 18]. EES was a second-generation DES, which was superior to BMS and first-generation DES in reducing the risk of stent thrombosis and repeat revascularization [1, 19]. Several trials compared DCB with EES for the treatment of ISR, but they had small sample sizes and inconsistent outcomes [4, 5, 8–11]. Thus, the clinical outcomes on the comparison of DCB and EES for the treatment of ISR were underpowered.

The present meta-analysis showed differential relative efficacy between DCB and EES in DES-ISR and BMS-ISR patients. DCB had comparable angiographic results and clinical events with EES in BMS-ISR patients. However, for DES-ISR patients, DCB was inferior to EES in terms of both angiographic results and clinical events. This situation has significant clinical implications. Currently, in the United States, more than 80% of stents implanted during PCI were DES [20]. In China, the proportion of DES use was up to 99.6% for stent implantation [21]. Even in patients with second-generation DES implantation, the incidence of ISR is higher than 10%, and the rate of repeat revascularization for DES-ISR is still encountered in 5–10% of patients undergoing percutaneous coronary intervention [22]. In other words, DES-ISR is the majority type of ISR in clinical practice, especially in China. Therefore, it is very important to clarify the treatment strategies of DES-ISR and BMS-ISR. In the latest European myocardial revascularization guidelines, both DES and DCB were recommended for the treatment of DES-ISR or BMS-ISR (Class I, Level A) [2]. The inferiority of DCB to EES for the treatment of DES-ISR in the present meta-analysis arisen a challenge for DCB treatment in DES-ISR patients and indicated expectation of more high-quality trials to further evaluate the efficacy of DCB for the treatment of DES-ISR.

Previous network meta-analysis showed that EES was more effective for the treatment of ISR compared with DCB, with the lowest risks of restenosis and repeat revascularization compared with other treatments. However, this network meta-analysis only include two head-to-head comparative trials, RIBS IV and RIBS V, to synthesize the direct result of EES versus DCB. Differed from the network meta-analysis, our study included six head-to-head trials, and the results from subgroup analyses indicated that EES was inferior to DCB only in DES-ISR, but not in BMS-ISR.

Previous studies had demonstrated that DCB angioplasty was more effective in BMS-ISR than in DES-ISR, with no difference on the type of DES [16, 23]. A prospective, multicenter, randomized trial conducted by Habara et al. on 208 patients showed that DCB reduced neointimal hyperplasia more effectively in BMS-ISR than in DES-ISR at 6 months angiographic and clinical follow-up after intervention [16]. SeQuent Please World Wide Registry showed that the TLR rate was significantly lower in patients with DCB angioplasty for BMS-ISR than in those for DES-ISR [23]. In the present study, the finding of differential relative efficacy for DCB between BMS-ISR and DES-ISR is consistent with the finding of these studies.

Several potential mechanisms are responsible for the varying efficacies of DCB on DES-ISR and BMS-ISR. First, the histomorphological features of neointimal differed in BMS-ISR and DES-ISR patients. Nakano et al. carried out a human autopsy registry and indicated that neointimal compositions of DES-ISR demonstrated greater proteoglycan deposition and less smooth muscle cellularity over time, compared with BMS-ISR with greater smooth muscle cell density and collagen deposition [24]. In the present meta-analysis, paclitaxel-coated balloons were used in all included trials. A previous study demonstrated that paclitaxel reduced in-stent intimal hyperplasia by inhibiting arterial smooth muscle cell proliferation and migration [25]. Therefore, the BMS-ISR with greater smooth muscle cell density might have higher drug efficacy. Second, DES-ISR might already have the drug resistance or local hypersensitivity reactions, whereas BMS-ISR is still naive regarding the treatment [26]. Third, ISR occurs earlier in patients implanted with BMS, and neointimal hyperplasia rich in smooth muscle cells is the prevalent mechanism [27]. While in patients with DES implantation, as the effects of antiproliferative drugs eluted by the stent impaired physiological reendothelialization and vascular remodeling, chronic inflammation, and delayed and aberrant arterial healing were frequently observed in-stent of DES, which induced the formation of neoatherosclerosis [27]. Delayed and abnormal arterial healing, persistent inflammatory process, and/or incompetent endothelial function might decrease the efficacy of DCB in DES-ISR patients [28].

This study had some potential limitations. First, paclitaxel-coated balloons were exclusively used DCB in all the included trials. Therefore, our findings only reflected the effect of paclitaxel-coated balloons for the treatment of ISR. Recently, a novel sirolimus-coated balloon was investigated in DES-ISR patients compared with paclitaxel-coated balloons [29]. The efficacy of the novel DCB for the treatment DES-ISR is still an ongoing exploration. Second, our findings need to be considered as average effects of DES-ISR, because detailed types of DES-ISR were not available in the present study. Given the similar mechanisms underlying different DES-ISR [27], the generalizability of our results seems viable. Third, while this type of analysis has particular shortcomings when discussing efficacy between EES and DEB such as different drug coatings, different stent platforms, and so on, the results of this analysis can be generalizable due to shared pathophysiology that contributes to in-stent restenosis. Fourth, consistent heterogeneity was observed for the angiographic results among included studies. Random-effects model was used to account for the heterogeneity. Fifth, the inherent limitations of meta-analyses cannot be ignored, such as publication bias.

## 5. Conclusions

DCB has differential relative efficacy between BMS-ISR and DES-ISR. In DES-ISR patients, DCB was associated with lower MLD, higher percent diameter stenosis, more binary stenosis, and higher rate of TVR and TLR. However, for BMS-ISR, no significant difference was found between DCB and EES. More high-quality randomized trials are needed to further evaluate the role of DCB for the treatment of ISR, especially in patients with DES-ISR. The potential mechanisms of the varying efficacy between BMS-ISR and DES-ISR for DCB need to be further investigated.

## Figures and Tables

**Table 1:** Baseline characteristics and Jadad scores of the included studies.

Study, year	Type of stents	Sample size		Age (y)		Male %		Hypertension, %		Diabetes, %		Previous MI		Previous CABG		Dyslipidemia		Smoking		Follow-up	
		DCB	EES	DCB	EES	DCB	EES	DCB	EES	DCB	EES	DCB	EES	DCB	EES	DCB	EES	DCB	EES	ACG, m	Clinical, y
DARE, 2018 [5]	DES and BMS	141	137	66 ± 11	65 ± 10	72%	84%	64%	67%	31%	33%	53%	52%	14%	16%	59%	60%	17%	13%	6	1
Restore, 2018 [4]	DES	86	86	67 ± 10	66 ± 9	70.9%	72.1%	69.8%	75.6%	50.0%	44.2%	30.2%	25.6%	NR	NR	NR	NR	46.5%	43.0%	9	1
RIBS IV, 2015 [9, 12]	DES	154	155	66 ± 10	66 ± 10	82.5%	83.9%	71%	78%	49%	43%	60%	60%	4%	7%	73%	66%	59%	75%^∗^	6–9	1
RIBS V, 2014 [11, 14]	BMS	95	94	67 ± 11	64 ± 12	86.3%	87.2%	72%	72%	32%	20%	47%	50%	10%	11%	71%	78%	58%	56%	6–9	1
SEDUCE, 2014 [10]	BMS	25	25	67.6 ± 7.7	64.2 ± 11	72%	100%	64%	60%	24%	4%	48%	40%	NR	NR	96%	96%	21%	12%	9	1
TIS, 2016 [6, 12]	BMS	68	68	65.6 ± 11	65.5 ± 11	63.2%	67.7%	NR	NR	25.0%	26.5%	63.2%	60.3%	4.4%	8.8%	NR	NR	45.6%	42.7%	12	1

Data are shown as percentage or mean ± standard deviation, unless otherwise stated. DCB, drug-coated balloons; EES, everolimus-eluting stents; DES, drug-eluting stents; BMS, bare-mental stents; MI, myocardial infarction; CABG, coronary artery bypass grafting; NR, not reported; ACG, angiography.

**Table 2:** Patients and lesion characteristics of the included studies.

Study	Group	Target vessel, %				RVD, mm	MLD, mm	DS%, %	LVEF, %	UA, %	SA/SI, %	ISR Mehran classification, %		
		LAD	LCX	RCA	VB							I	II	III-IV
DARE, 2018 [5]	DCB	41.0%	0.0%	37.0%	0.7%	2.56 ± 0.43	0.77 ± 0.33	69.7 ± 11.8	NR	44%	NR	51%	32%	17%
	EES	39.0%	0.7%	35.0%	1.4%	2.59 ± 0.54	0.79 ± 0.35	69.3 ± 12.5	NR	42%	NR	53%	34%	13%
RESTORE, 2018 [4]	DCB	55.8%	15.1%	27.9%	1.2%	2.85 ± 0.50	0.63 ± 0.40	77 ± 17	59.4% ± 8.4%	45.30%	41.80%	67.20%	14.90%	17.90%
	EES	60.5%	12.8%	24.4%	0.0%	3.06 ± 0.45	0.63 ± 0.42	79 ± 13	59.9% ± 7.8%	38.40%	45.40%	66.20%	19.10%	14.70%
RIBS IV, 2015 [9, 12]	DCB	37.0%	22.0%	39.0%	2.0%	2.64 ± 0.60	1.02 ± 0.40	61 ± 14	58% ± 13%	40%	60%	63%	34%	3%
	EES	39.0%	23.0%	34.0%	3.0%	2.64 ± 0.60	0.93 ± 0.40	65 ± 13	59% ±1 2%	45%	56%	64%	28%	8%
RIBS V, 2014 [11, 14]	DCB	50.0%	18.0%	28.0%	4.0%	2.58 ± 0.50	0.79 ± 0.40	69 ± 17	58% ± 12%	52%	48%	40%	47%	13%
	EES	46.0%	22.0%	29.0%	3.0%	2.55 ± 0.50	0.75 ± 0.40	72 ± 15	59% ± 11%	51%	49%	36%	45%	19%
SEDUCE, 2014 [10]	DCB	24.0%	20.0%	52.0%	4.0%	3.00 ± 0.48	0.98 ± 0.60	67.7 ± 18.4	NR	20%	76%	32%	52%	16%
	EES	44.0%	28.0%	24.0%	0.0%	2.85 ± 0.44	0.57 ± 0.37	79.4 ± 13.5	NR	20%	76%	36%	40%	24%
TIS, 2016 [6, 12]	DCB	47.3%	NR	29.7%	1.4%	2.64 ± 0.47	0.92 ± 0.45	71.8 ± 13.9	49.7% ± 12.0%	NR	64.70%	40.54%	45.95%	13.52%
	EES	54.1%	NR	29.7%	2.7%	2.66 ± 0.45	0.79 ± 0.48	78.0 ± 13.4	49.6% ± 11.4%	NR	63.20%	28.38%	47.30%	24.32%

Data are shown as percentage or mean ± standard deviation. DCB, drug-coated balloons; EES, everolimus-eluting stents; LAD, anterior descending branch; LCX, left circumflex artery; RCA, right coronary artery; VB, vein bypass; RVD, reference vessel diameter; MLD, minimum lumen diameter; DS, diameter stenosis; LVEF, left ventricular ejection fraction; UA, unstable angina; SA/SI, stable angina/silent ischemia; ISR, in-stent stenosis; NR, not reported.

**Table 3:** Summary of the main results.

Items	Population					
	*N*1	DES-ISR: pooled effect size DCB:EES (95% CI)	*N*2	BMS-ISR: pooled effect size DCB:EES (95%CI)	*N*(*N*1 + *N*2)	Overall: pooled effect size DCB:EES (95%CI)
MLD	481	*MD* = −0.25(−0.37, − 0.14)^∗^	375	*MD* = −0.15(−0.39, 0.09)	1134	*MD* = −0.17(−0.29, − 0.05)^∗^
LLL	481	*MD* = 0.04(−0.12, 0.20)	375	*MD* = −0.06(−0.35, 0.24)	1134	*MD* = −0.06(−0.23, 0.10)
Percent diameter stenosis	481	*MD* = 7.45(3.80, 11.09)^∗^	375	*MD* = 4.69(−4.98, 14.36)	1134	*MD* = 5.37(1.33, 9.42)^∗^
Binary restenosis	481	*RR* = 2.07(1.20, 3.58)^∗^	375	*RR* = 0.89(0.47, 1.68)	1134	*RR* = 0.77(0.44, 1.34)
TLR	481	*RR* = 2.43(1.28, 4.62)^∗^	239	*RR* = 2.23(0.70, 7.10)	720	*RR* = 2.38(1.36, 4.18)^∗^
TVR	481	*RR* = 2.07(1.22, 3.50)^∗^	375	*RR* = 0.82(0.46, 1.46)	1134	*RR* = 1.17(0.52, 2.61)
Myocardial infarction	481	*RR* = 1.01(0.22, 4.73)	375	*RR* = 0.80(0.31, 2.06)	1134	*RR* = 0.91(0.48, 1.73)
Death	481	*RR* = 1.10(0.50, 2.41)	375	*RR* = 1.45(0.62, 3.36)	1134	*RR* = 1.19(0.68, 2.07)

DCB, drug-coated balloons; EES, everolimus-eluting stents; DES, drug-eluting stents; BMS, bare-mental stents; MLD, minimum lumen diameter; LLL, late lumen loss; TLR, target lesion revascularization; TVR, target vessel revascularization; MD, mean difference; RR, risk ratio; CI, confidence interval. ^∗^*P* < 0.05.

## Abbreviations

DCB: drug-coated balloons
EES: everolimus-eluting stents
ISR: in-stent restenosis
DES: drug-eluting stents
BMS: bare-mental stents
MLD: minimum lumen diameter
TVR: target vessel revascularization
TLR: target lesion revascularization
LLL: late lumen loss.
